# Molecular mechanisms involved in alcohol craving, IRF3, and endoplasmic reticulum stress: a multi-omics study

**DOI:** 10.1038/s41398-024-02880-5

**Published:** 2024-03-26

**Authors:** Ming-Fen Ho, Cheng Zhang, Irene Moon, Mustafa Tuncturk, Brandon J. Coombes, Joanna Biernacka, Michelle Skime, Tyler S. Oesterle, Victor M. Karpyak, Hu Li, Richard Weinshilboum

**Affiliations:** 1 Department of Psychiatry and Psychology, Mayo Clinic, 200 First Street SW, Rochester, MN 55905 USA; 2 Department of Molecular Pharmacology and Experimental Therapeutics, Mayo Clinic, 200 First Street SW, Rochester, MN 55905 USA; 3 Department of Health Sciences Research, Mayo Clinic, 200 First Street SW, Rochester, MN 55905 USA

**Keywords:** Molecular neuroscience, Stem cells

## Abstract

Alcohol use disorder (AUD) is the most prevalent substance use disorder worldwide. Acamprosate and naltrexone are anti-craving drugs used in AUD pharmacotherapy. However, molecular mechanisms underlying their anti-craving effect remain unclear. This study utilized a patient-derived induced pluripotent stem cell (iPSC)-based model system and anti-craving drugs that are used to treat AUD as “molecular probes” to identify possible mechanisms associated with alcohol craving. We examined the pathophysiology of craving and anti-craving drugs by performing functional genomics studies using iPSC-derived astrocytes and next-generation sequencing. Specifically, RNA sequencing performed using peripheral blood mononuclear cells from AUD patients with extreme values for alcohol craving intensity prior to treatment showed that inflammation-related pathways were highly associated with alcohol cravings. We then performed a genome-wide assessment of chromatin accessibility and gene expression profiles of induced iPSC-derived astrocytes in response to ethanol or anti-craving drugs. Those experiments identified drug-dependent epigenomic signatures, with IRF3 as the most significantly enriched motif in chromatin accessible regions. Furthermore, the activation of IRF3 was associated with ethanol-induced endoplasmic reticulum (ER) stress which could be attenuated by anti-craving drugs, suggesting that ER stress attenuation might be a target for anti-craving agents. In conclusion, we found that craving intensity was associated with alcohol consumption and treatment outcomes. Our functional genomic studies suggest possible relationships among craving, ER stress, IRF3 and the actions of anti-craving drugs.

## Introduction

Alcohol use disorder (AUD) is the most prevalent substance use disorder worldwide [1]. Alcohol craving is an essential symptom of AUD, according to the Diagnostic and Statistical Manual for Mental Disorders, 5th edition (DSM-5), and it is associated with alcohol relapse [2–4]. Craving is a subjective drive that has proven challenging to define and measure precisely. The Penn Alcohol Craving Scale (PACS) is one of the most widely used self-reported questionnaires used to evaluate craving in AUD clinical research [5, 6]. However, there currently is little agreement on standard measures of craving in clinical practice.

Disulfiram, acamprosate, and naltrexone have been approved in the United States by the FDA to treat AUD. Disulfiram discourages alcohol use by causing severe side effects when alcohol is consumed [7]. Therefore, it is only recommended for AUD patients who can be monitored closely and are motivated to abstain from alcohol use [8]. Acamprosate and naltrexone are frequently described as anti-craving drugs [9, 10]. Naltrexone is a µ opioid receptor antagonist and an anti-craving drug that has received FDA approval for the pharmacotherapy of AUD and opioid use disorder [11]. Naltrexone affects neurotransmission, neuroinflammation, and the hypothalamic-pituitary-adrenal axis [12, 13]. However, the molecular mechanism of its anti-craving effects remains unclear. Acamprosate is a compound with a chemical structure similar to the neurotransmitter GABA and the amino acid taurine [14]. Most medical literature on AUD pharmacotherapy and acamprosate mechanism(s) of action has emphasized its effects on the balance between GABAergic inhibitory and glutamatergic excitatory effects in the brain [15]. Acamprosate does not undergo metabolism and is excreted unchanged in the urine [16, 17]. It has been reported that acamprosate helps balance disrupted neurotransmission by decreasing overexcitation induced by alcohol [18]. However, like naltrexone, acamprosate’s molecular mechanism(s) of action as a treatment for AUD remains unclear. To better understand the mechanisms by which naltrexone and acamprosate alleviate cravings, we designed the present study to use these medications as “molecular probes”. We joined these probes with AUD patient-derived cell line model systems to identify genes that might be associated with craving intensity which is known to contribute to variation in treatment outcomes.

Previously, we conducted an acamprosate clinical trial. Patients with AUD (*n* = 442) were treated with acamprosate for three months in community-based treatment programs [19, 20]. We collected comprehensive clinical information for this study cohort before and after three months of acamprosate treatment. The primary outcome of the clinical trial was acamprosate treatment response [21, 22]. We previously identified alcohol craving as the most significant clinical phenotype associated with treatment outcomes [23, 24]. Specifically, higher baseline craving intensity was associated with relapse to alcohol use during the three months of acamprosate treatment [22, 23, 25]. A recent study suggested that astrocytes displayed a higher number of differentially expressed genes (DEGs) in the prefrontal cortex of alcohol-dependent subjects compared to control donors [26]. Current in vitro assays and in vivo models designed to study the pathophysiology of cravings are limited [27]. Therefore, using AUD patient-derived induced pluripotent stem cells (iPSCs) and brain cell types differentiated from those iPSCs as model systems represents a “cell-line based” approach to advance our understanding of both drug action and AUD disease pathophysiology. Therefore, the present study was designed to evaluate the molecular mechanisms associated with alcohol craving and anti-craving agents using an iPSC-based cell model system. We set out to 1) compare gene expression profiles between high and low craving intensity AUD patients, and 2) identify molecular signatures for ethanol (EtOH) and for AUD anti-craving drugs in iPSC-derived astrocytes.

## Materials and Methods

### Ethics statements

We conducted this study under protocols reviewed and approved by the Mayo Clinic Institutional Review Board (IRB numbers: 07-007204, 18-006428, and 20-00372). All participants provided informed consent. We maintained confidentiality for all study participants.

### iPSC-based cell model system

We generated a panel of six iPSCs from AUD patients (Supplementary Table 1). Specifically, we utilized peripheral blood mononuclear cells (PBMCs) for iPSC reprogramming using the CytoTune™-iPS 2.0 Sendai Reprogramming Kit (A16517, Thermo Fisher, USA). AUD patient-derived iPSCs were then characterized as previously described [21, 28]. Briefly, iPSCs were cultured on Matrigel-coated plates (BD Biosciences) in mTeSR1 medium (STEMCELL technology, MA, USA). All iPSCs were regularly verified to be free from mycoplasma. All cell lines revealed normal karyotypes, and they all expressed pluripotency markers. AUD patient-derived iPSCs (*n* = 6, Supplementary Table 1) were differentiated into astrocytes [28–30]. Briefly, iPSC colonies were detached from the Matrigel (Corning) with 1 mg ml^−1^ collagenase treatment for an hour and the cells were suspended in embryoid body (EB) medium, consisting of FGF-2-free iPS cell medium supplemented with 2 μM dorsomorphin (Sigma) and 2 μM A-83 (Sigma), in non-treated polystyrene plates for six days. The medium was changed daily. After six days, we replaced the EB medium with neural induction medium (hNPC medium) consisting of DMEM/F12, N2 supplement, NEAA, 2 μg ml^−1^ heparin (Tocris Bioscience) and 2 μM cyclopamine (Tocris Bioscience). Floating EBs were then transferred to Matrigel-coated 6-well plates at day 7 to form neural tube-like rosettes. The attached rosettes were kept for 15 days with hNPC medium change every other day. The rosettes were transferred and cultured in low attachment plates in hNPC medium containing B27 (Thermo-Fisher) On day 22. Neural progenitor spheres were then dissociated with Accutase (STEMCELL technology, MA, USA) on day 24, and placed onto Matrigel-coated plates in astrocyte culture medium (1801, ScienCell) [28]. Cells were prepared for subsequent experiments between passages 3 and 6.

### Drug treatment

We treated cells with EtOH (25 mM). This concentration is considered physiologically relevant for EtOH use, with 25 mM EtOH being slightly higher than the 0.08% blood alcohol concentration that is often used as a measure of intoxication [31]. The concentrations of acamprosate (5 µM, Sigma, A6981) and naltrexone (30 nM, Selleckchem, S2103) used to perform those experiments were selected to fall within the range of blood concentrations for these drugs observed during clinical therapy [32]. Cells were cultured with the drugs for seven days and the medium was changed daily.

### RNA sequencing and data analysis

Peripheral blood mononuclear cells (PBMCs) samples were isolated from whole blood using Ficoll density gradient centrifugation. We lysed cells in Trizol, and extracted total RNA using the RNeasy mini kit (Qiagen, Valencia, CA, USA). The RNA integrity numbers (RIN) were 8.5-9.2 for the 12 PBMC samples. We also performed RNA-seq using iPSC-derived astrocytes (*n* = 6) before and after drug exposure (EtOH, acamprosate, and naltrexone). The RIN numbers were above 9 for all RNA samples from iPSC-derived astrocytes. RNA-seq experiments were conducted by GENEWIZ using an Illumina HiSeq 4000 with eight samples in each lane using 100 bp paired end index reads (Supplementary Table 2). We aligned fastq files containing paired RNA-Seq reads with STAR [33] against the UCSC human reference genome (hg19). We performed RNA-seq differential expression analysis using the DESeq2 package with default parameters [34]. The significance threshold (FDR < 0.05) was applied to identify differentially expressed genes (DEGs). These values were adjusted using the Benjamini-Hochberg method to correct for multiple hypothesis testing. We used gene set enrichment analysis (GSEA) software to perform pathway analysis [35, 36].

### Assay for transposase accessible chromatin using sequencing (ATAC-seq)

The ATAC-seq was performed using iPSC-derived astrocytes (*n* = 3) before and after drug exposure (EtOH, acamprosate, and naltrexone). ATAC-seq experiments were conducted by GENEWIZ using Illumina HiSeq 2 × 150 bp sequencing, single index, paired-end platform (Supplementary Table 2). MACS2 software was used for peak calling, R package DiffBind was used to determine differential peaks for each drug treatment condition vs vehicle treatment. Motif discovery analyses were performed using the analysis of motif enrichment (AME) from MEME suite (https://meme-suite.org/meme/doc/ame.html). Linear regression was performed using ATAC-seq tag density and IRF3 ChIP-seq data (GSE91752) [37].

### Immunofluorescence staining and confocal imaging analysis

iPSC-derived astrocytes were grown on glass coverslips. Cells were then fixed in 4% paraformaldehyde at room temperature for 15 min. The cells were permeabilized with 0.2% Triton X-100 in PBS. After blocking for 30 min with 3% normal donkey serum in PBS, cells were incubated with primary antibody in 5% BSA (see Supplementary Table 3) overnight. The secondary antibody was used at a 1:1000 dilution. We used Antifade mounting media with DAPI (VECTOR laboratory, Burlingame, CA, USA) to stain the cell nuclei. We visualized slides using fluorescence microscopy (Olympus, FV1200).

### Western blot analysis

Protein samples were isolated from iPSC-derived astrocytes. The membranes were incubated overnight with primary antibodies (Supplementary Table 3) at 4°C. We then incubated the washed membranes with anti-rabbit or anti-mouse secondary antibodies at room temperature for an hour. The washed membranes were subsequently incubated in Pierce^®^ ECL Western blotting substrate (Thermo Scientific, Madison, WI, USA) and were visualized using Geldoc (Biorad, Hercules, CA, USA).

### Chromatin immunoprecipitation (ChIP) assays

ChIP assays were performed with iPSC-derived astrocytes using the MAGnify Chromatin Immunoprecipitation System (Invitrogen, CA, USA). DNA-IRF3 complexes were immunoprecipitated using antibodies directed against IRF3 (IRF-3 (D83B9) Rabbit mAb #4302, Cell signaling technology, Danvers, MA, USA) with rabbit IgG as a control. After purification, DNA was subjected to qPCR using the primer sets listed in Supplementary Table 3. Primer efficiency was optimized by a forward and reverse primer concentration gradient from 0.5 μM to 2.5 mM. The amplification efficiency was measured using 5-fold serial dilution (1/20, 1/100, 1/500, 1/2500, 1/12500, and 1/62500) of cDNA template in duplicated measurements. The exponential amplification efficiency (E) value should be approximately 2. This value is calculated using the equation: E = 10^(-1/slope)^. If the slope is −3.32, the PCR reaction will reach 100% efficiency, as the PCR product exactly doubles during each cycle [38]. The primer efficiency data are listed in Supplementary Table 3.

$${\bf{E}}={{\bf{10}}}^{(-{\bf{1}}/-{\bf{3.32}})}={\bf{2}}={\bf{100}} \%\, {\bf{efficiency}}$$

We determined the percentage of ChIP DNA/input by real time PCR. We expressed the level of enrichment (percent of input) as relative enrichment above background (enrichment relative to IgG control).

### ER stress assay

The cell stress assays (Green cell stress sensor, #U0900G, Montana Molecular, Boxeman, MT) can detect cellular stress in living cells. This cell stress assay is packaged in BacMam, a BSL-1 viral vector. The cell stress assay is nuclear targeted and mimics the endogenous regulatory pathway of the XBP1 protein which is the sensor on which the assay is based. This assay produces a bright green fluorescent protein when the cells experience ER stress or undergoes the unfolded protein response, i.e. XBP1 has been shown to be ethanol inducible in iPSC-derived astrocytes. We used this assay as a readout for ER stress in a scalable and quantitative manner. The Cell Stress Assay Kit was used to determine the cell stress in three replicates. Cells were seeded (5000 cells/well) in 96-well poly-D lysine coated plates. Green fluorescence was measured using a microplate reader with excitation at 485 nm and emission detection at 528 nm. Data were normalized to control wells (untransduced cells). The fold change in green fluorescence was used to compare cell stress in response to the various treatments that were studied.

### Statistical analysis

We performed statistical analysis using R Statistical Software (version 4.0.5; R Foundation for Statistical Computing, Vienna, Austria). ChIP-qPCR results were analyzed using ANOVA, followed by Tukey’s multiple comparison tests for individual comparisons when significant effects were detected. *P* < 0.05 was considered statistically significant.

## Results

Our previous studies suggested that craving intensity (*p*: 9.36E-06) was the most significant predictor of acamprosate treatment outcome [23, 24]. We set out in the present experiments to study the craving pathophysiology and anti-craving drugs’ effect. We began this series of studies by determining genome-wide gene expression profiles for peripheral blood mononuclear cells (PBMCs) obtained from AUD patients with extreme values for baseline PACS scores (30 vs 0). Baseline clinical information and blood samples were collected after enrollment and before acamprosate treatment [20, 21]. We also determined genome-wide RNA expression profiles of human iPSC-derived astrocytes in response to treatment with EtOH, acamprosate, naltrexone or vehicle (PBS). Pathway analysis of those genome-wide expression data placed a focus on immune-related pathways. In addition, we observed a striking gene expression pattern in which a large number of expression signals for EtOH and the anti-craving drugs used in AUD pharmacotherapy were anti-correlated. This series of observations, taken as a whole, emphasized possible relationships between craving and inflammation and the effect of anti-craving drugs on inflammation. Finally, we identified IRF3 as a transcription factor that plays a role in ethanol-induced endoplasmic reticulum (ER) stress—an effect that can be attenuated by anti-craving drugs. As a result, the findings described subsequently may help to enhance our understanding of the mechanism(s) of action of anti-craving medications and provide novel insight into the pathophysiology of craving.

### Identification of genes associated with alcohol craving in AUD patients

**Fig. 1:**
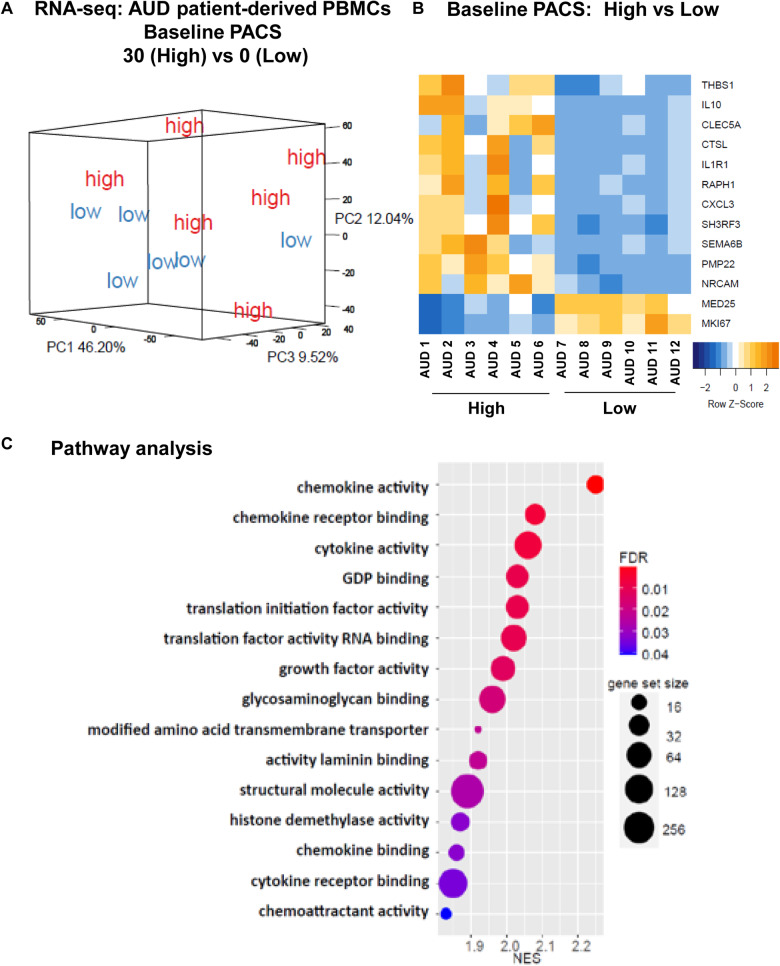
Effects of alcohol craving on gene expression profiles in AUD patients. **A** Principal components analysis (PCA) of gene expression profiles in PBMCs obtained at baseline from AUD patients with extreme values for baseline PACS scores. Specifically, six subjects with an average baseline PACS score of 0 (low), and six subjects with an average baseline PACS score of 30 (high) were selected for PBMC RNA-seq. **B** A heatmap plot showing expression profiles for the most DEGs between high craving and low craving intensity groups based on the PBMC RNA-seq data (FDR < 0.05). **C** Pathway analysis of the PBMC RNA-seq data was performed using gene set enrichment analysis (GSEA) software [35, 36]. NES is the normalized enrichment score to account for the size of each gene set.

The PACS is one of the most commonly used assessments for alcohol cravings [5, 39]. The PACS is a five-item self-report craving scale. Each question has a score from 0-6. Therefore, the maximum total score is 30. However, there is no established cutoff PACS score to determine the risk of relapse or the “severity” of craving. As a result, we turned to biological assays and performed RNA-seq using PBMCs obtained at baseline from AUD patients with extreme values for baseline PACS scores (Supplementary Fig. 1). We had six subjects with a baseline PACS score of 0 (low) and six subjects with a baseline PACS score of 30 (high), all of whom had baseline PBMC samples available for RNA-seq (clinical characteristics of the 12 subjects are listed in Supplementary Table 1). Specifically, alcohol consumption measures for 30 days or 90 days prior to enrollment revealed no significant difference between low and high craving subjects (Supplementary Table 1). However, principal component analysis (PCA) of gene expression profiles showed distinct clustering for the two groups, i.e. the low PACS score group versus the high PACS score group (Fig. 1A). The heatmap plot in Fig. 1B shows results for the most significant differentially expressed genes (DEGs) between these two groups with FDR < 0.05 (full results in Supplementary Table 4). The genes shown in Fig. 1B will be discussed in the subsequent functional genomic study. We also observed that the largest number of significant DEGs involved immune response related pathways (see Fig. 1C). These observations are consistent with an extensive literature reporting that activation of immune response signaling is an important biological feature of AUD pathology [40]. It should also be pointed out that most of these genes were upregulated in AUD patients with higher baseline alcohol craving intensity. Therefore, we hypothesized that EtOH and anti-craving drugs might regulate genes related to alcohol craving intensity as shown in Fig. 1B.

### Anti-correlated gene expression patterns after ethanol treatment or anti-craving drug exposure

**Fig. 2:**
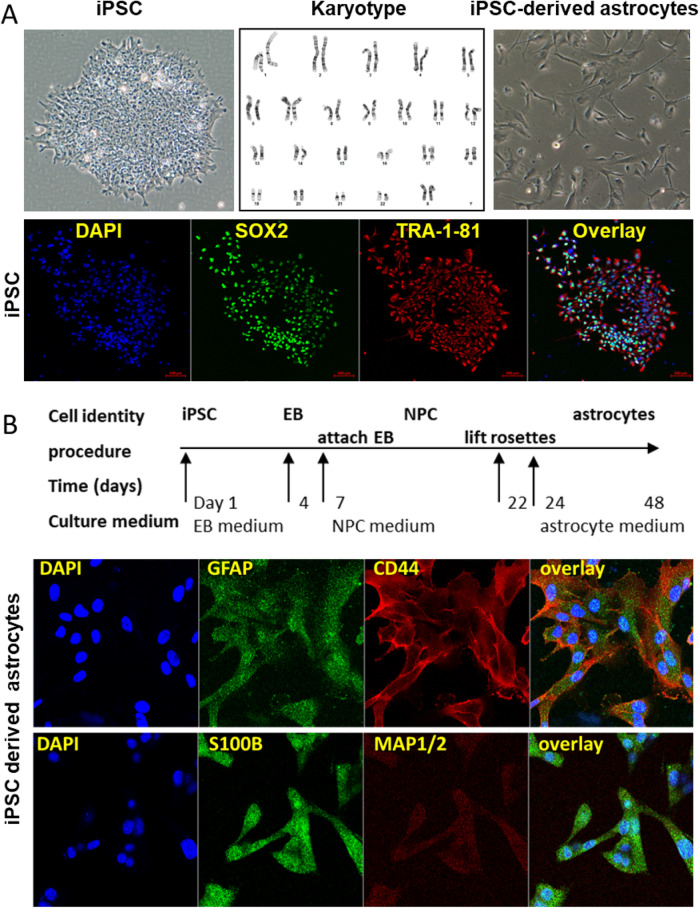
Generation and characterization of iPSC-derived astrocytes. **A** iPSCs from six AUD patients were generated from peripheral blood mononuclear cells (PBMCs). Those cells all showed normal karyotypes as well as the expression of pluripotency markers. This panel displays representative data for iPSC cells from AUD patients with the three panels showing iPSCs in culture, an example of a normal karyotype, and iPSC-derived astrocytes. **B** A schematic outline for procedures used in the differentiation of iPSC-derived astrocytes. The panel below the schematic displays representative examples of staining for astrocyte markers (GFAP, CD44 and S100β). MAP1/2 is a marker of neurons.

**Fig. 3:**
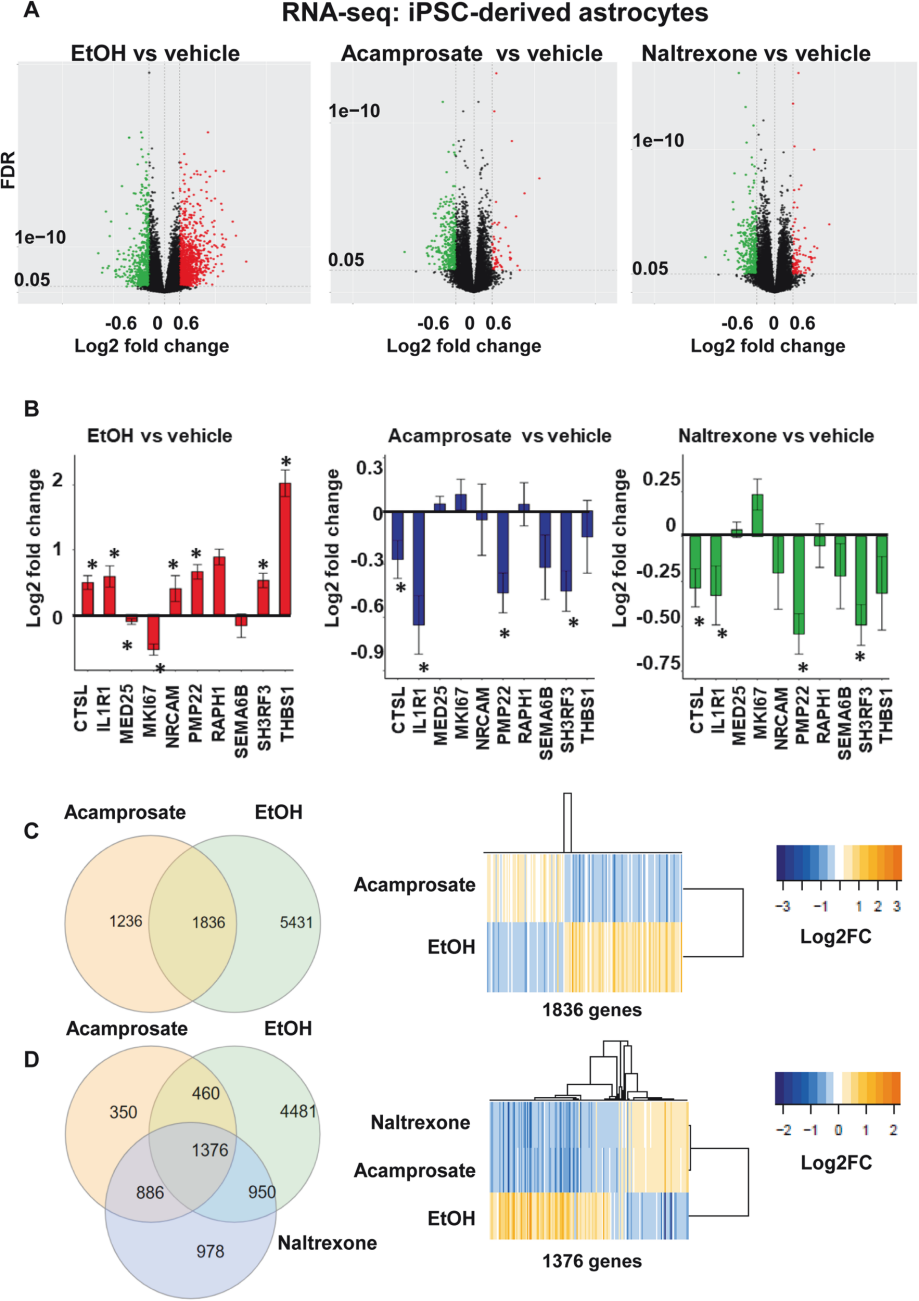
Gene expression profiles of iPSC-derived astrocytes in response to treatment with EtOH, acamprosate or naltrexone. **A** The volcano plots show differential gene expression profiles after exposure to EtOH (25 mM), acamprosate (5 µM) or naltrexone (30 nM) for 7 days, respectively, as compared to vehicle treatment. RNA-seq was performed using iPSC-derived astrocytes from six AUD subjects. Two biological replicates were performed for each sample. **B** Effects of EtOH, acamprosate or naltrexone in iPSC-derived astrocytes on expression of genes encoding inflammatory mediators, as determined by RNA-seq. *FDR < 0.05. **C** Left panel: Venn diagram showing the 1836 genes with expression that was affected by both EtOH and acamprosate as determined by RNA-seq (FDR < 0.05). Right panel: Heatmap showing expression profiles for the 1836 genes were affected by both EtOH and acamprosate. Note that the expression of these genes was “anti-correlated” when results after EtOH and acamprosate treatment were compared. **D** Left panel: Venn diagram showing expression profiles for the 1376 genes with expression that could be affected by EtOH, acamprosate, and naltrexone as determined by RNA-seq (FDR < 0.05). Right panel: Heatmap showing expression profiles for the 1376 genes affected by EtOH, acamprosate and naltrexone. Note that the expression of these genes was “anti-correlated” when comparing results after EtOH and after anti-craving drug exposure.

We designed the next series of experiments to explore the effects of EtOH on the genes associated with alcohol craving with regulation that was altered by EtOH and by anti-craving drugs in iPSC-derived astrocytes (Fig. 2). All iPS cell lines revealed normal karyotypes, and they all expressed pluripotency markers (Fig. 2A). AUD patient-derived iPSCs were differentiated into astrocytes (Fig. 2B). Astrocytes were used in these studies because a recent study suggested that astrocytes displayed the highest number of DEGs in the prefrontal cortex of alcohol-dependent subjects compared to control donors [26]. In addition, iPSC-derived astrocytes are immunocompetent and can respond to inflammatory stimuli and sustain inflammation by producing pro-inflammatory cytokines, similar to the behavior of primary astrocytes [41]. As mentioned previously, several immune related pathways are associated with elevated alcohol craving intensity (Fig. 1C). Therefore, we set out to perform RNA-seq for iPSC-derived astrocytes from six AUD subjects in response to exposure to EtOH, acamprosate, or naltrexone for seven days. Volcano plots showed 7267, 3072, and 4190 genes with expression significantly altered (FDR < 0.05) after exposure to EtOH, acamprosate, or naltrexone treatment, respectively, as compared to vehicle treatment (Fig. 3A with full results in Supplementary Table 5). Specifically, most genes associated with high alcohol craving, as shown in Fig. 1B were ethanol responsive in iPSC-derived astrocytes (Fig. 3B). We also determined whether acamprosate might regulate this same panel of genes. In the presence of acamprosate, the gene expression pattern was “inverted”. In other words, the genes with expression elevated after EtOH exposure showed decreased expression after acamprosate and vice versa (Fig. 3B middle panel). Similar results were observed after exposure to naltrexone (Fig. 3B right panel). These observations indicated that EtOH and anti-craving drugs could influence gene expression in distinct and opposite directions. They also suggested the possibility of using EtOH and the anti-craving agents for the pharmacotherapy of AUD as “molecular probes” to identify and study genes that might be related to alcohol craving in a genome-wide fashion by performing RNA-seq. As anticipated, there was a significant overlap of the genes affected by both EtOH and acamprosate with FDR < 0.05 (Fig. 3C left panel). Remarkably, we observed a striking gene expression pattern, in which the expression of a large number of the overlapping genes displayed “anti-correlated gene expression patterns” when comparing results after EtOH and acamprosate treatment (Fig. 3C right panel). Acamprosate and naltrexone have distinct gene expression profiles. However, they might also have some shared therapeutic biology. Specifically, we observed a significant overlap (1376 genes) of DEGs in response to treatment with EtOH, acamprosate, and naltrexone, with FDR < 0.05 (Fig. 3D left panel). Even more striking, we once again observed a “mirror image” with regard to the direction of gene expression patterns for EtOH and both of the anti-craving drugs (Fig. 3D right panel).

Furthermore, we identified a series of immune-related pathways associated with genes affected by drug treatment. Remarkably, the list of immune-related pathways displayed opposite directionality for changes in expression between EtOH and the anti-craving drugs—as shown by the normalized enrichment score (NES) values listed in Supplementary Table 6. These results were also compatible with the RNA-seq data for PBMCs obtained from AUD subjects (see Fig. 1C), which indicated that enrichment for genes in immune-related pathways was associated with elevated alcohol craving intensity. The next set of experiments was designed to study molecular mechanisms underlying the gene expression regulation pattern of the ethanol responsive genes associated with alcohol craving.

### Chromatin accessible regions contain the IRF3 motif and display IRF3 binding

**Fig. 4:**
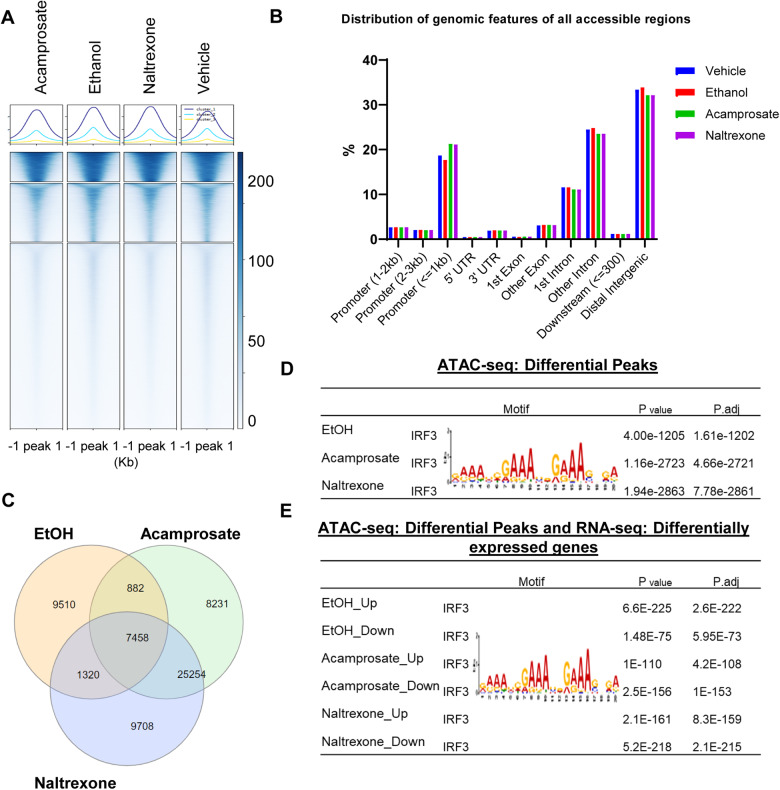
Chromatin accessibility profiling of iPSC-derived astrocytes in response to treatment with EtOH, acamprosate or naltrexone. **A** ATAC-seq was performed using iPSC-derived astrocytes (*n* = 3) with or without drug treatment i.e. EtOH (25 mM), acamprosate (5 µM) or naltrexone (30 nM) for 7 days. Peaks were clustered by K-means clustering. The numbers on the right indicate peak signal density. **B** Distribution of genomic features of genome-wide DNA accessible regions. **C** Venn diagram showing chromatin accessible regions that could be affected by EtOH, acamprosate and naltrexone individually, paired, or all three as determined by ATAC-seq (FDR < 0.05). **D** Motif discovery analyses were performed using differential ATAC-seq peaks (FDR < 0.05) for each drug treatment condition. IRF3 was the most significant transcription factor enriched in the differential peak regions across all three treatment conditions. **E** Motif discovery analyses were performed using differential peaks as determined by ATAC-seq (FDR < 0.05) and DEGs as determined by RNA-seq (FDR < 0.05) after each drug treatment condition. Up=increase in both chromatin accessibility and gene expression. Down=decrease in both chromatin accessibility and gene expression.

Chromatin accessibility broadly reflects transcriptional regulation capacity. We began this series of studies by performing assays for transposase accessible chromatin using sequencing (ATAC-seq). These results could potentially help us uncover the genomic architecture and transcription factors (TF) responsible for the transcriptional regulation of the anti-craving drugs used to treat AUD. The genome-wide chromatin accessibility signals across all four treatment conditions are shown in Fig. 4A. We observed a similar distribution of genomic features for all accessible regions between the different treatment conditions (Fig. 4B and Supplementary Fig. 2). To identify specific dynamic changes in the chromatin accessibility landscape in response to exposure to EtOH or anti-craving drugs, we next analyzed the differentially accessible peaks (Fig. 4C). Specifically, the most significantly enriched motif identified in the differentially accessible peaks was the IRF3 binding motif when the cells were exposed to EtOH. Surprisingly, the same TF was identified when cells were exposed to acamprosate or naltrexone (Fig. 4D, the full list can be seen in Supplementary Table 7). However, those results did not explain the anti-correlated gene expression pattern in response to ethanol, and the anti-craving drugs, as shown in Fig. 3D.

We then integrated the RNA-seq data with the ATAC-seq data coupled with TF motif analysis in an attempt to identify transcriptional networks that contributed to gene expression regulation during drug exposure (Fig. 4E). Once again, we found that IRF3 was the top hit for the motif discovery analysis in the presence of EtOH (Fig. 4E, the full list is presented in Supplementary Table 8). The same TF was also identified after exposure to the anti-craving drugs (Fig. 4E). These results suggest that IRF3 might be able to bind to the DEGs in response to drug treatment.

### IRF3 binding and regulation of genes associated with alcohol craving

**Fig. 5:**
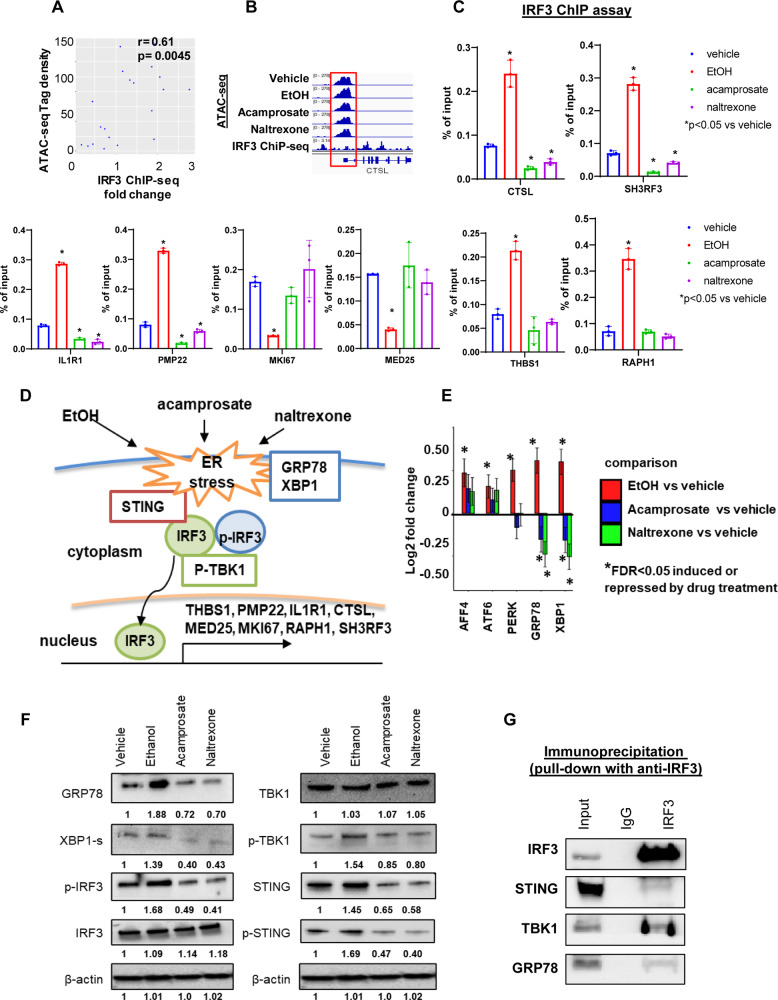
IRF3 is a transcription factor which can regulate the expression of genes associated with alcohol craving and the activation of IRF3 is associated with ethanol-induced endoplasmic reticulum (ER) stress signaling which can be attenuated by anti-craving drugs. **A** ATAC-seq tag density was correlated positively with IRF3 binding density in the promoter regions of the genes associated with alcohol craving as shown in Fig. 1B. The dot plot represents basal ATAC peak quantification (y axis) and IRF3 ChIP-seq fold change over input (x axis). **B** Representative regional plot in the CTSL locus showing open chromatin tracks in iPSC-derived astrocytes in response to EtOH (25 mM), acamprosate (5 µM) or naltrexone (30 nM) treatment of iPSC-derived astrocytes and IRF3 ChIP-seq (GSE91752) [37]. Note that the ATAC-seq tracks represent ATAC-seq tag density, and the publically available IRF3 ChIP-seq track represents IRF3 binding fold change over control. **C** ChIP assays showing the effect of IRF3 binding to the promoter regions of genes that were associated with alcohol craving intensity in response to EtOH (25 mM), acamprosate (5 µM) or naltrexone (30 nM) treatment of iPSC-derived astrocytes (*n* = 3). Percentage of ChIP DNA/input was determined by qPCR. Data are represented as % input, (enrichment relative to IgG control) = % input (IRF3 antibody) - % input (IgG). One-way ANOVA was used for data analysis. *P < 0.05 vs vehicle. Three independent experiments were performed. **D** Cartoon model for the activation of IRF3 and endoplasmic reticulum (ER) stress induced by ethanol. ER stress genes, ie GRP78 and XBP-1, can be induced by ethanol. STING phosphorylation can recruit IRF3, which itself can be phosphorylated by TBK1. This process will enhance IRF3 translocation and activate the transcription of downstream genes. Specifically, phospho-TBK1—a kinase required for IRF3 phosphorylation, and STING—an adaptor protein that resides in the ER membrane--were also induced by ethanol. However, exposure to anti-craving drugs i.e. acamprosate or naltrexone, could attenuate ER stress signaling. IRF3 protein directly interacted with STING, TBK1, and GRP78. As a result, IRF3 might play a role in ethanol-induced ER stress through the phosphorylation of IRF3. This, in turn, could facilitate the translocation of IRF3 to the nucleus as a transcription factor which plays a role in the regulation of gene expression. **E** mRNA expression of ER stress genes in response to exposure to ethanol, acamprosate or naltrexone in iPSC-derived astrocytes as determined by RNA-seq (*n* = 6). *FDR < 0.05. **F** Protein expression of GRP78, XBP-1s, p-IRF3, IRF3, TBK1, pTBK1, STING, and p-STING in iPSC-derived astrocytes in response to exposure to ethanol, acamprosate or naltrexone was determined by Western blot analysis (*n* = 4). Alpha-tubulin and vinculin were used as loading controls. Images are representative of iPSC-derived astrocytes from AUD patients (*n* = 4). **G** Immunoprecipitation was used to determine whether IRF3 protein could interact with STING, TBK1 and GRP78 in iPSC-derived astrocytes. Whole cell lysates from 1×10^7^ iPSC-derived astrocytes were immunoprecipitated with anti-IRF3 (1:50) antibodies or anti-IgG antibodies. Pull down protein samples were immunoblotted and probed with antibodies against IRF3, STING, TBK1 and GRP78 (**D**). Pulldowns are representative of iPSC-derived astrocytes from four AUD subjects.

We consulted the ENCODE database [42] and determined whether IRF3 binding sites were correlated with chromatin accessible regions. We observed a significant positive correlation between chromatin accessibility and IRF3 binding in the promoter regions of DEGs in a genome-wide fashion (Supplementary Fig. 3A). We next performed IRF3 ChIP assays using iPSC-derived astrocytes to verify IRF3 binding, focusing on genes associated with alcohol craving, as shown in Fig. 1B and Fig. 3B. Specifically, Fig. 5A and Supplementary Fig. 3B show that IRF3 binding density was highly correlated with ATAC-seq tag density for genes associated with alcohol craving, as shown in Fig. 1B, all of which have IRF3 binding sites in promoters of the genes that map to open chromatin accessible regions (Fig. 5B and Supplementary Fig. 4). Furthermore, ChIP assays (Fig. 5C) showed that EtOH exposure induced IRF3 binding for all of the genes except *MED25* and *MKI67*. However, in the presence of acamprosate or naltrexone, IRF3 binding decreased significantly for *CSTL, SH3RF3, IL1R1*, and *PMP22*, consistent with the gene expression results shown in Fig. 3B. We were able to replicate these findings as shown in Fig. 5C and demonstrate the specific IRF3 binding in the promoter region of those genes in another panel of iPSC-derived astrocytes (see Supplementary Fig 5). Of importance, IRF3 expression was not altered in response to either ethanol or anti-craving drugs. The subsequent paragraph describes how IRF3 was activated as a transcription factor, with effects that could alter the expression of downstream genes in a drug-dependent manner.

### Knockdown of IRF3 suppressed ethanol-induced endoplasmic reticulum (ER) stress

**Fig. 6:**
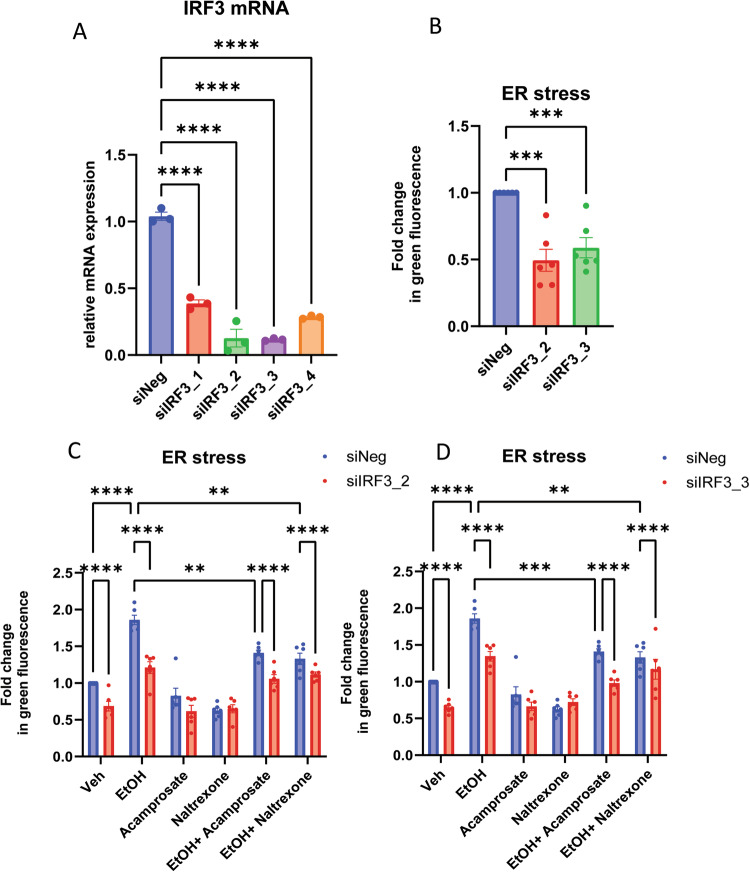
The effects of IRF3 on ER stress in iPSC-derived astrocytes. **A** We tested IRF3 siRNA knockdown efficiency and found that among the four individual IRF3 siRNA, siIRF3_2 and siIRF3_3 can knock down IRF3 to ~10% of its baseline (F (4, 10) = 120.2, *p* < 0.0001). We then chose those two siRNA for the subsequent experiments. **B** Knockdown of IRF3 in iPSC-derived astrocytes resulted in decrease ER stress (*n* = 6) F (2, 15) = 17.39, *p* = 0.0001. **C** ER stress assays were performed in iPSC-derived astrocytes exposed to vehicle, ethanol (EtOH), acamprosate, naltrexone, ethanol+acamprosate, and ethanol+naltrexone, with and without IRF3 Knockdown using siIRF3_2 (*n* = 6, the full list of comparison made are presented in Supplementary Table 9). **D** We used another IRF3 siRNA (siIRF3_3) to confirm our findings as shown in (**C**) (the full list of comparison made are presented in Supplementary Table 10). siNeg scrambled siRNA.

It is well-documented that alcohol can induce ER stress and the phosphorylation of IRF3 [43]. STING phosphorylation can recruit IRF3, which can be phosphorylated by TBK1 and which then forms a homodimer (Fig. 5D). As a result, IRF3 enters the nucleus and activates the transcription of downstream genes, including many of the genes that we found to have expression that was altered by exposure to EtOH, acamprosate, and naltrexone (Fig. 5D) [43, 44]. To explore this possibility, we consulted our RNA-seq data and found that a series of ER stress genes, i.e. GRP78, XBP1, PERK, AFF4, and ATF6, were ethanol inducible. However, only GRP78 and XBP1 expression could be down-regulated by acamprosate and naltrexone (Fig. 5E). We also observed that IRF3 activation was induced in iPSC-derived astrocytes by EtOH through the phosphorylation of IRF3 (Fig. 5F). In addition, phospho-TBK1—a kinase required for IRF3 phosphorylation, and STING—an adaptor protein that resides in the ER membrane, were also induced by EtOH (Fig. 5F). Furthermore, when the cells were treated with acamprosate or naltrexone, we found opposite effects, i.e. decreased phosphorylation of IRF3, TBK1, and STING (Fig. 5F). It has been reported that STING-IRF3 pathways are associated with ER stress and that IRF3 binds to TBK1 and STING in hepatocytes [43]. In line with those observations, our results showed that IRF3 protein directly interacts with STING, TBK1, and GRP78 in iPSC-derived astrocytes (Fig. 5G). These observations support the hypothesis that IRF3 activation in iPSC-derived astrocytes might play a role in EtOH-induced ER stress through the phosphorylation of IRF3. That, in turn, could facilitate the translocation of IRF3 to the nucleus as a transcription factor playing a role in the expression of the genes that we found to be influenced by exposure to EtOH, acamprosate and naltrexone (see Figs. 1B, 3B). To further strengthen our findings, we knocked down IRF3 in iPSC-derived astrocytes using siRNA and found that IRF3 suppressed ER stress (Fig. 6A, B). In addition, ethanol could induce ER stress, however, in the absence of IRF3, ethanol-induced ER stress was attenuated (Fig. 6C). Furthermore, both acamprosate and naltrexone treatment decreased ER stress. We then took one step further to test the effects of the combination of ethanol and anti-craving drugs on ER stress. As anticipated, ethanol-induced ER stress can be attenuated by anti-craving drugs. Lower ER stress was observed in IRF3 knockdown cells treated with anti-craving drugs, as compared to cells transfected with scrambled siRNA and treated with ethanol plus acamprosate or naltrexone. We confirmed those results using two individual IRF3 siRNA (Fig. 6C, D, the full list of comparison made are presented in Supplementary Tables 9 and 10).

## Discussion

Elevated craving intensity has been associated with an increased probability of alcohol relapse among AUD patients [45]. This study was designed to use AUD patient-derived cell-line model systems and FDA approved anti-craving drugs used in the pharmacotherapy of AUD as “molecular probes” to identify possible mechanisms associated with craving intensity, an important factor that contributes to AUD treatment outcomes.

To identify possible molecular mechanisms involved in alcohol craving, we performed RNA-seq using PBMC samples from AUD patients with extreme PACS scores. Notably, the 12 baseline PBMC samples from our AUD patients with extreme PACS scores did not show significant differences in baseline alcohol consumption (Supplementary Table 1). As a result, the differences in baseline PBMC expression profiles between the two groups are unlikely to be related to recent exposure to alcohol. We should point out that all subjects were required to maintain abstinence before medication started [21, 22]. We did not measure blood alcohol concentration at the time of enrollment [22]. However, we used self-report Timeline Follow Back (TLFB) to track alcohol use from 90 days before enrollment to 90 days after initiating acamprosate treatment [20, 24]. Our results suggest that inflammation might play a role in alcohol cravings. Similar to our observations, several previous studies have reported that cytokines such as IL8 and IL1β were positively correlated with alcohol consumption and alcohol craving intensity [46–48]. Furthermore, a randomized, crossover, double-blind, placebo-controlled study reported that ibudilast, an anti-inflammatory agent, reduced craving in AUD patients [49]. Those observations highlight the possibility that decreasing craving intensity by reducing inflammation might help reduce the probability of alcohol relapse.

Our studies used ethanol and anti-craving drugs as molecular probes to determine gene expression profiles in iPSC-derived astrocytes. We observed that the expression of genes in immune-related pathways was changed significantly after drug or ethanol exposure. In parallel, a recent study showed that astrocytes and microglia displayed distinct transcriptome alterations in the mouse prefrontal cortex following chronic intermittent ethanol vapor exposure. Of importance, that study identified ~800 genes in the prefrontal cortex of mouse models which could be regulated by ethanol exposure [50], and ~50% of those genes were replicated in our human iPSC-derived astrocytes. Similar to our observations, that study also demonstrated that type I interferon signaling was consistently involved in changes in gene expression in astrocytes, microglia, and total homogenate [50]. These findings further strengthen the conclusion that ethanol and anti-craving drugs might play a role in the type I interferon signaling pathway. Acamprosate and naltrexone have distinct mechanisms of action with different molecular targets. Naltrexone is a µ opioid receptor antagonist. However, it should be pointed out that opioid receptors are not expressed in iPSC-derived astrocytes. Therefore, our results suggest that the anti-inflammatory effects of naltrexone do not appear to be dependent on opioid receptor activity. It has been reported that low-dose naltrexone may have anti-inflammatory effects on several chronic conditions i.e. chronic pain and Crohn’s disease [51]. It has also been reported that naltrexone can decrease the production of pro-inflammatory cytokines such as IL6 and TNFα by PBMCs following stimulation with ligands for a series of toll-like receptors (TLR), including TLR7, TLR8, and TLR9 [52]. That study supports our findings that naltrexone could have anti-inflammatory effects in the absence of opioid receptors.

Integration of RNA-seq and ATAC-seq made it possible to determine TF “footprints” which might help to define the biology underlying alcohol craving. Specifically, we identified potential TFs within differential chromatin accessible regions that might be associated with regulating gene expression after drug exposure. We suspected the possible involvement of IRF3 in our iPSC-derived astrocytes based on the location of candidate motifs identified in our motif discovery studies and the location of chromatin accessible regions. Specifically, IRF3 acts as a transcription factor that can influence the expression of a series of genes that we identified as associated with alcohol craving. It is known that ER stress promotes IRF3 phosphorylation, which in turn, can influence innate immune signaling and cytokine production [43, 53, 54]. Furthermore, it is well-documented that alcohol can induce ER stress in several organs, including the brain [55]. Acamprosate has been reported to have anti-inflammatory effects [56, 57], and naltrexone can attenuate inflammation and ER stress-induced liver injury [58]. Our results are compatible with the hypothesis that ethanol can induce the activation of IRF3 via ER stress which, in turn, can alter the expression of downstream genes associated with alcohol craving. However, these effects can be reversed by anti-craving drugs, suggesting that an effect on ER stress signaling might be one of the mechanisms of action for the anti-craving agents we studied.

Our study also has limitations. First, we performed functional genomic studies using iPSC-derived astrocytes, a cell type implicated in AUD’s pathophysiology [59, 60]. However, iPSC-derived cell lines, like all cell lines, have limitations. Future studies that include different brain cell types or 3-D brain organoids will be required to pursue the results reported here. We failed to generate iPSCs from the patients for whom we have RNA-seq results in PBMCs as shown in Fig. 1B because the cell viability was too low after iPSC reprogramming. Our experiments were performed using only a single concentration and a single time point for each treatment condition. It might add additional value if we could have included a combination treatment i.e. ethanol plus acamprosate or naltrexone. However, that would also add another layer of data complexity that will raise questions beyond this study’s scope. However, even considering these limitations, the results reported here represent a potentially important step in obtaining functional insight into molecular mechanisms of action for the anti-craving drugs used clinically to treat AUD.

In conclusion, craving intensity is associated with alcohol consumption, and craving intensity appears to be the most significant predictor of alcohol relapse [23, 24]. We identified a series of genes enriched in immune-related pathways associated with elevated baseline craving intensity, which, as mentioned previously, can contribute to treatment response. We then demonstrated that those genes could be regulated by ethanol in iPSC-derived astrocytes. We also observed a striking gene expression pattern, in which a large number of expression signals displayed anti-correlated gene expression profiles with those for ethanol and the anti-craving drugs used in AUD pharmacotherapy. Finally, we demonstrated that IRF3 is a transcription factor which plays a role in ethanol-induced ER stress—an effect that can be attenuated by anti-craving drugs, suggesting that ER stress signaling might be one of the targets for anti-craving agents. This series of observations could represent an important step toward advancing our understanding of the pathophysiology of both craving and mechanisms of anti-craving drug action.

### Supplementary information

Supplementary data legends

Supplementary Figures

Supplementary Tables

## Data Availability

All data supporting our findings can be found in the main paper or in supplementary files. Sequencing data are available via the GEO accession number: GSE213380.
