# Supplementary material for: Molecular mechanisms involved in alcohol craving, IRF3, and endoplasmic reticulum stress: a multi-omics study
Source: Transl Psychiatry. 2024 Mar 26;14:165. doi: 10.1038/s41398-024-02880-5 (PMC10965952; doi:10.1038/s41398-024-02880-5)
Supplement: Supplementary file 1 — Supplementary data legends [file 41398_2024_2880_MOESM1_ESM.docx]

# Molecular mechanisms involved in alcohol craving, IRF3, and endoplasmic reticulum stress: a multi-omics study

# Ming-Fen Ho, Ph.D.^1,2*^, Cheng Zhang, Ph.D.^2^, Irene Moon, MS^2^, Mustafa Tuncturk, M.D.^1^, Brandon J. Coombes, Ph.D.^3^, Joanna Biernacka, Ph.D.^3^, Michelle Skime, M.S.^1^, Tyler S. Oesterle, M.D. M.P.H.^1^, Victor M Karpyak, M.D., Ph.D.^1^, Hu Li, Ph.D.^2^, and Richard Weinshilboum, M.D.^2*^

***Corresponding authors:**

Ming-Fen Ho, Ph.D., Mayo Clinic 200 First Street SW, Rochester, MN 55905,

Phone: 507-293-3387, Email: ho.mingfen@mayo.edu

Richard Weinshilboum, M.D., Mayo Clinic 200 First Street SW, Rochester, MN 55905,

Phone: 507-284-2790, Email: richard.weinshilboum.mayo.edu

**Extended  Data Legends**

**Supplementary Fig 1.** PACS score distribution for the PBMC samples obtained at baseline from AUD patients. We performed RNA-seq using six subjects with baseline PACS scores of 0 (low), and six subjects with baseline PACS scores of 30 (high).

**Supplementary Fig 2.** Distribution of genomic features of genome-wide DNA accessible regions for all samples.

**Supplementary Fig 3.** (A) ATAC-seq tag density was correlated positively with IRF3 binding density in the promoter regions of the differentially expressed genes (DEGs) in response to treatments. The dot plot represents basal ATAC peak quantification (y axis) and IRF3 ChIP-seq fold change over input (x axis). (B) ATAC-seq tag density correlated positively with IRF3 binding density in the promoter regions of the genes associated with alcohol craving as shown in **Figure 2B**. The dot plots represent basal ATAC peak quantification (y axis) and IRF3 ChIP-seq fold change over the input (x axis).

**Supplementary Fig 4.** Regional plots showing open chromatin tracks in iPSC-derived astrocytes (n=3) in response to EtOH (25 mM), acamprosate (5 µM) or naltrexone (30 nM) treatment of iPSC-derived astrocytes, and IRF3 ChIP-seq (GSE91752). Red arrows indicate transcription start sites (TSS).

**Supplementary Fig 5.** IRF3 is a transcription factor which can regulate the expression of genes associated with alcohol craving. (A) Immunostaining for astrocyte markers (GFAP (green) and CD44 (red)). We generated iPSC-derived astrocytes from an additional 6 patients with AUD. Those cells were used for IRF3 ChIP assays as shown in panel B. (B) ChIP assays were performed to confirm the occupancy of IRF3 on selected binding regions as shown in Figure 5C. ChIP assay results showing the effect of IRF3 binding to the promoter regions of genes that were associated with alcohol craving intensity in response to EtOH (25 mM), acamprosate (5 µM) or naltrexone (30 nM) treatment of iPSC-derived astrocytes (n=6 AUD subjects). Percentage of ChIP DNA/input was determined by qPCR. Data are represented as % input, (enrichment relative to IgG control) = % input (IRF3 antibody) - % input (IgG). One-way ANOVA was used for data analysis. *P<0.05 vs vehicle. (C) A representative agarose gel photo illustrates IRF3 ChIP-qPCR amplified specific PCR products for eight selected genes as shown in panel B.

**Supplementary Table 1**. Characteristics of AUD patient-derived cell lines.

**Supplementary Table 2**. RNA-seq data and ATAC-seq quality control metrics for iPSC-derived astrocytes from AUD patients (n=6). A: acamprosate. E: ethanol. V: vehicle. N: naltrexone.

**Supplementary Table 3**. Primer sequences and antibodies used for experiments.

**Supplementary Table 4**. RNA-seq of PBMC samples from AUD patients: six subjects with an average baseline PACS score 0 (low), and six subjects with an average baseline PACS score 30 (high).

**Supplementary Table 5.** RNA-seq of iPSC-derived astrocytes from 6 AUD patients. A: acamprosate; E: ethanol; N: naltrexone; V: vehicle.

**Supplementary Table 6**. Pathway analysis of genes for which expression was altered in response to EtOH, acamprosate or naltrexone treatment in iPSC-derived astrocytes as determined by RNA-seq data. NES: normalized enrichment score to account for the size of each gene set. NES reflects the extent that the gene set is overrepresented at the top (positive NES) or bottom (negative NES) of the entire gene list ranked by log2 fold change. An FDR of <0.05 is deemed statistically significant. Size: number of gene count in each pathway.

**Supplementary Table 7**. Motif discovery analyses were performed using differential ATAC-seq peaks (FDR<0.05, drug treatment vs vehicle) in each drug treatment condition. IRF3 is the most significant transcription factor that was enriched in the differential peak regions across all three treatment conditions.

**Supplementary Table 8**. Motif discovery analyses were performed using differential peaks as determined by ATAC-seq (FDR<0.05) and differentially expressed genes as determined by RNA-seq (FDR<0.05) in each drug treatment condition. UP: increase both chromatin accessibility and gene expression. Down: decrease both chromatin accessibility and gene expression.

**Supplementary Table 9**. Statistical analysis for ER stress assays as shown in Fig. 6C.

**Supplementary Table 10**. Statistical analysis for ER stress assays as shown in Fig. 6D.
