# Supplementary material for: Molecular mechanisms involved in alcohol craving, IRF3, and endoplasmic reticulum stress: a multi-omics study
Source: Transl Psychiatry. 2024 Mar 26;14:165. doi: 10.1038/s41398-024-02880-5 (PMC10965952; doi:10.1038/s41398-024-02880-5)

Supplementary Fig 1

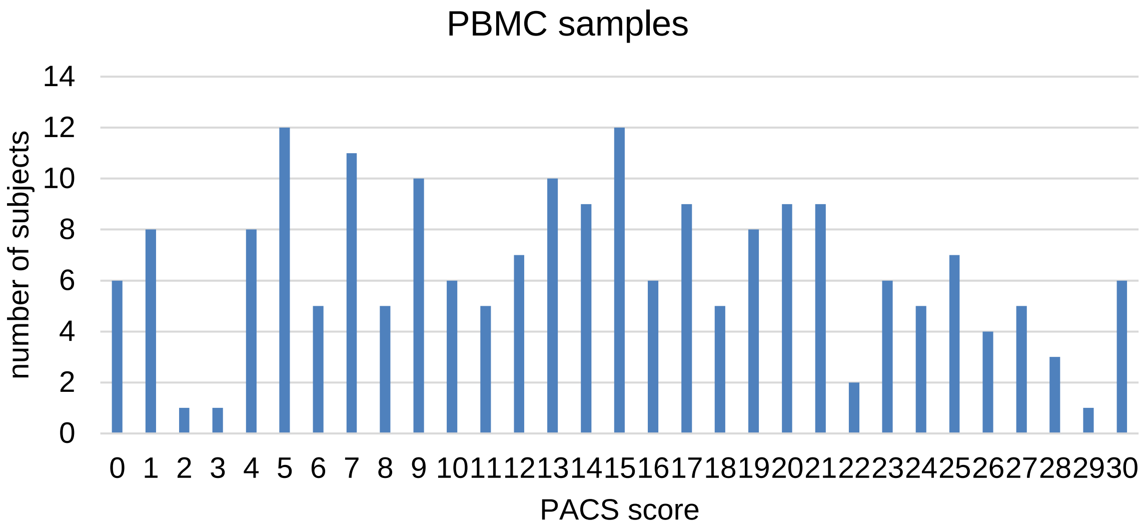

Supplementary Fig 2

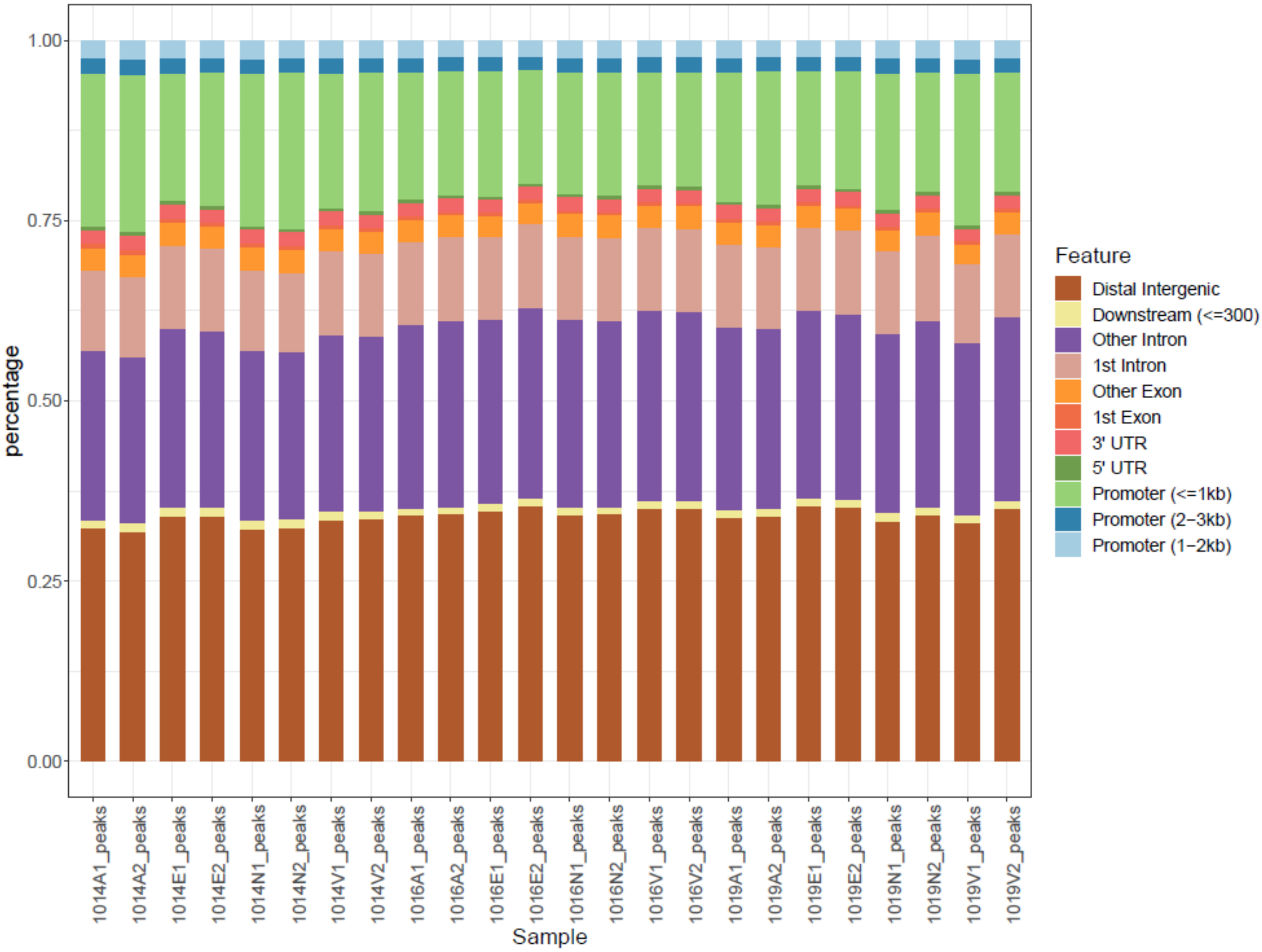

Supplementary Fig 3

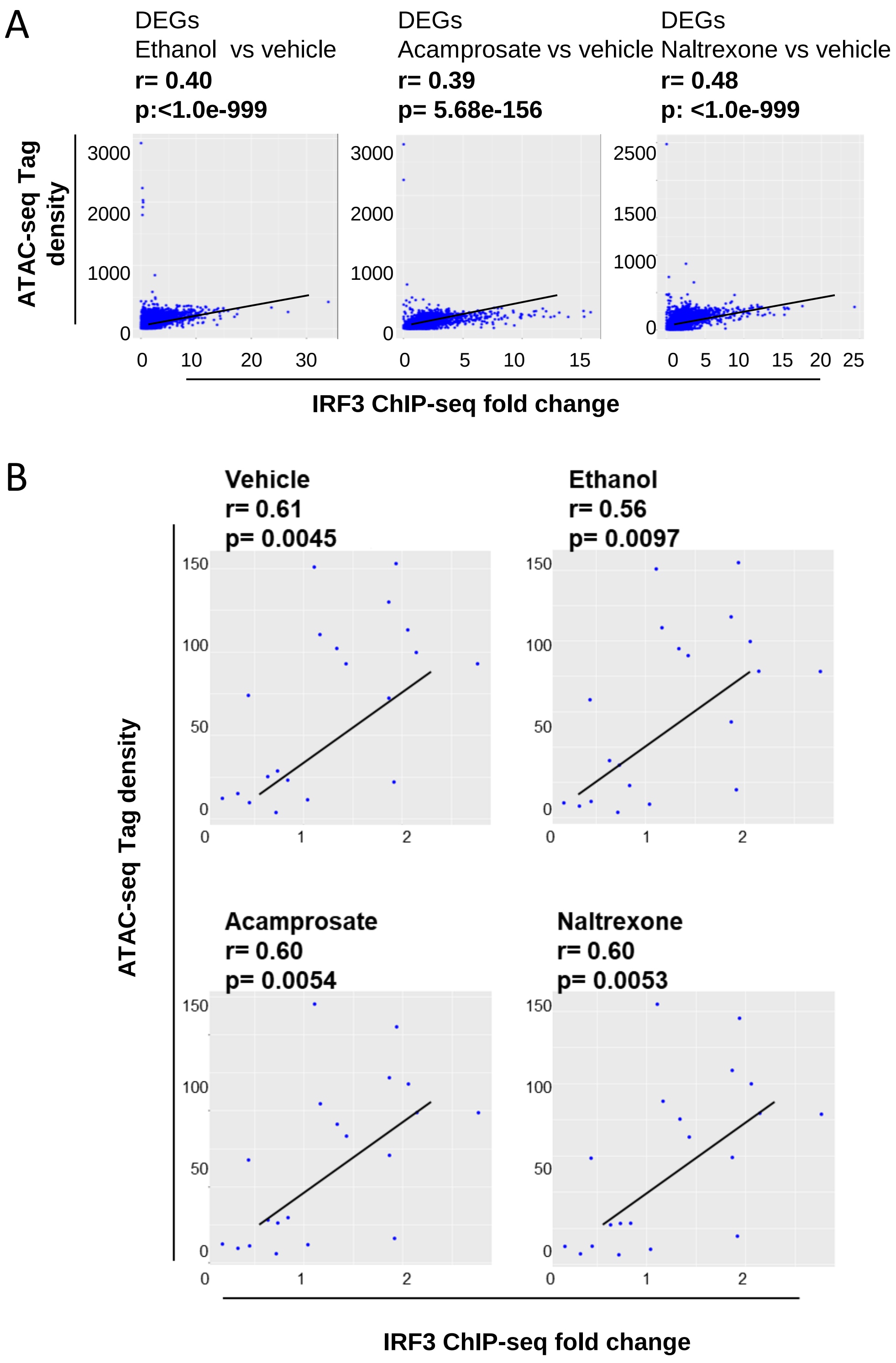

Supplementary Fig 4

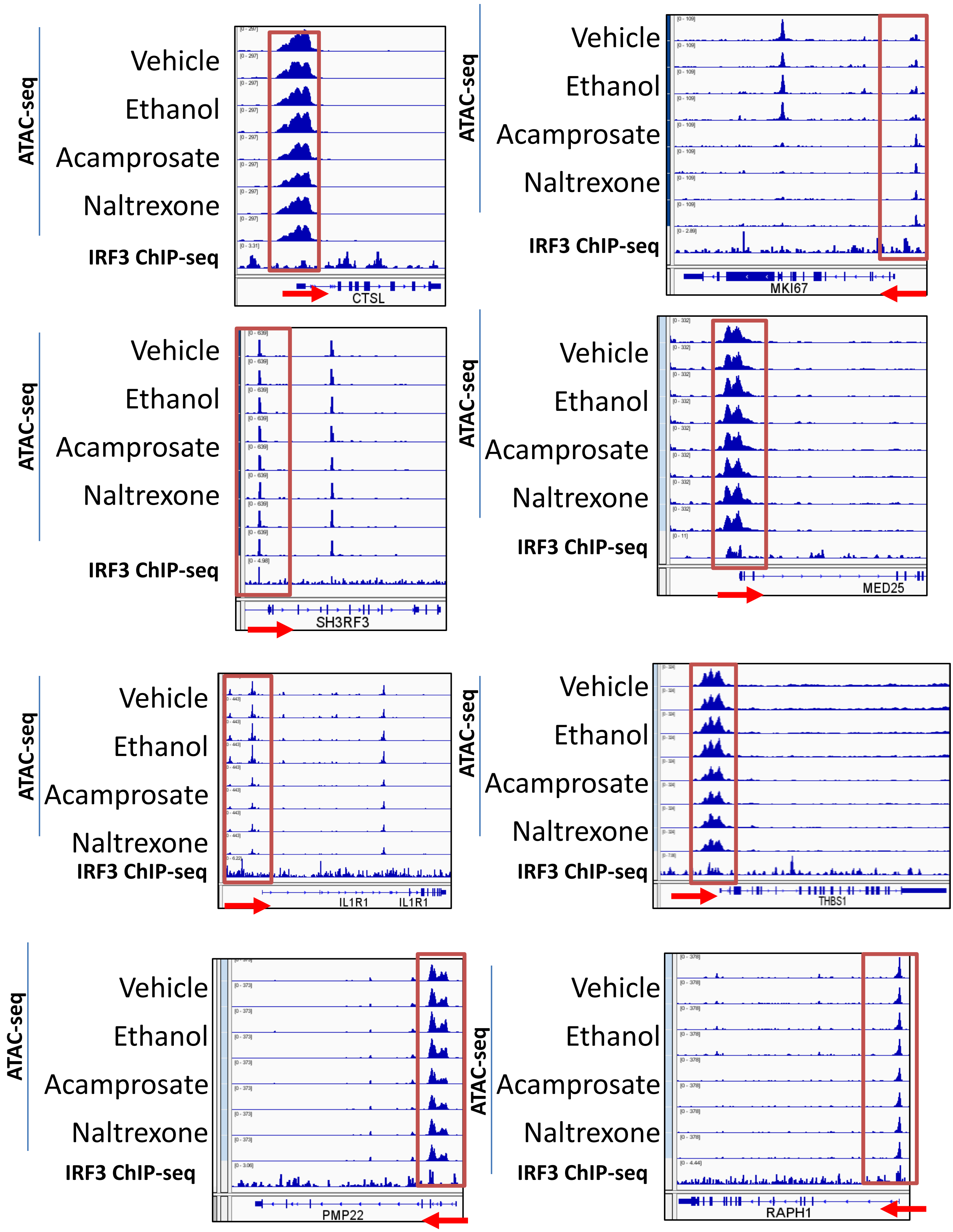

Supplementary Fig 5

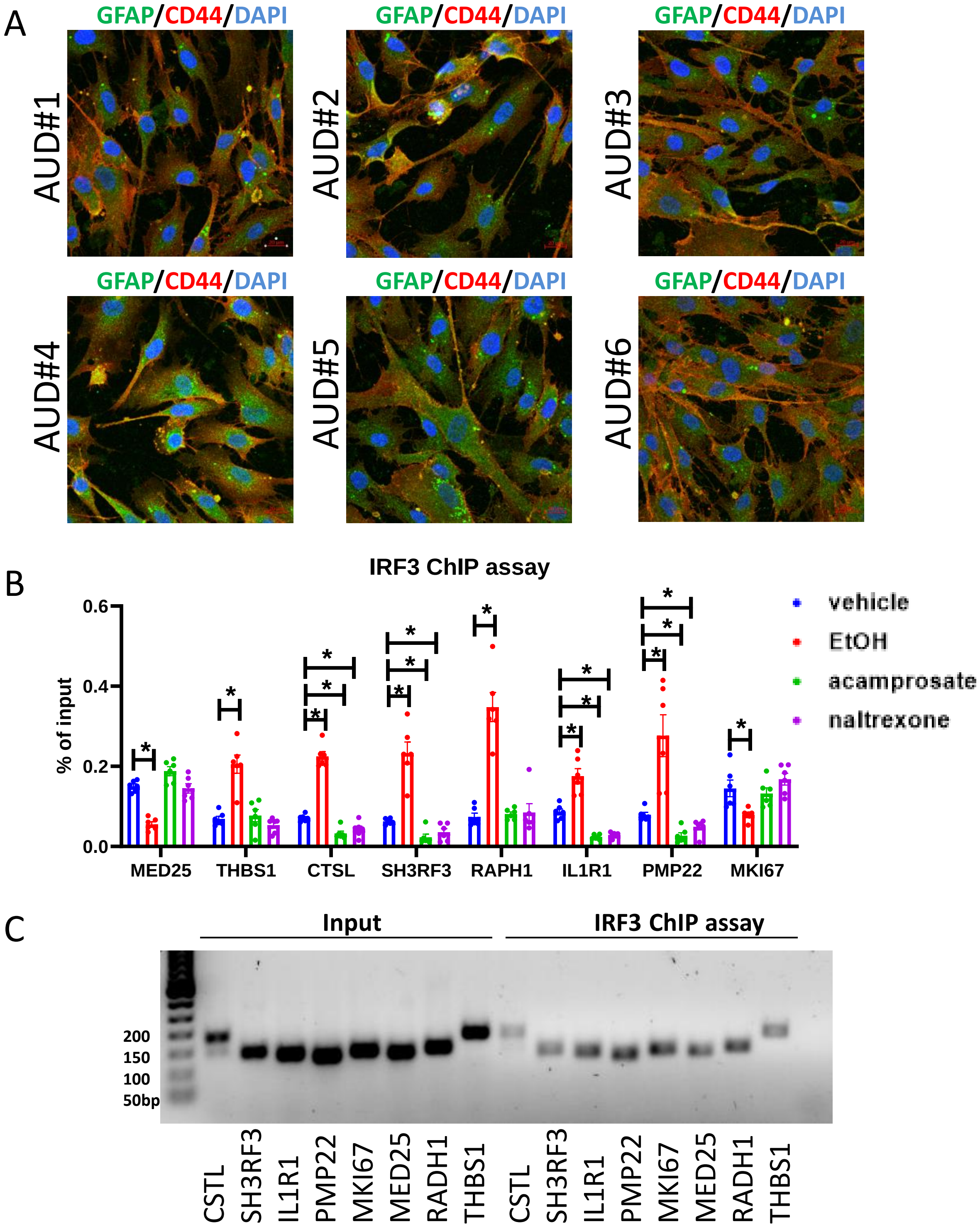

Supplement: Supplementary file 2 — Supplementary Figures [file 41398_2024_2880_MOESM2_ESM.pdf]
